# The Second Hit: Delayed Pulmonary Embolism After Minor Trauma and Initially Negative Imaging

**DOI:** 10.7759/cureus.91765

**Published:** 2025-09-07

**Authors:** Rahini Ranganathan, Sushma Thapa, Mahmoud Abughazal, Pemba Tamang, Girirajan Rajan

**Affiliations:** 1 Emergency Medicine, United Lincolnshire Hospitals NHS Trust, Boston, GBR; 2 Emergency Medicine, Pilgrim Hospital, United Lincolnshire Hospitals NHS Trust, Boston, GBR

**Keywords:** ct pulmonary angiogram (ctpa), elevated d-dimer, post-trauma, pulmonary embolism (pe), shortness of breath (sob)

## Abstract

Pulmonary embolism (PE) is a potentially fatal condition, typically associated with known risk factors such as immobility or thrombophilia. This case describes a 55-year-old woman with cardiomegaly who developed PE one week after sustaining minor chest trauma, despite an initially normal D-dimer and CT scan. She later re-presented with worsening respiratory symptoms, and a CT pulmonary angiogram confirmed PE. She was successfully treated with anticoagulation. This case highlights the need for ongoing clinical vigilance for PE, even in low-risk patients with initially unremarkable findings, particularly when symptoms evolve. It demonstrates that even minor chest trauma can precipitate a thromboembolic event and emphasizes that a normal initial assessment should not exclude the possibility of PE if clinical status deteriorates. Furthermore, it underlines the importance of timely reassessment with repeat imaging in the presence of evolving symptoms and highlights that early recognition and prompt initiation of anticoagulation are essential for improving patient outcomes.

## Introduction

Pulmonary embolism (PE) is a life-threatening condition characterized by obstruction of the pulmonary arteries, most commonly due to thrombi originating from the deep veins of the lower limbs or pelvis [1]. It is a major cause of cardiovascular morbidity and mortality worldwide, with an estimated annual incidence of 60-70 per 100,000 population in Europe and North America [2]. Although the pathophysiology of PE is well established and typically associated with known risk factors, such as recent surgery, immobility, malignancy, or inherited thrombophilia, cases arising without these traditional triggers remain diagnostically challenging [3]. Trauma, particularly severe or polytrauma, has been recognized as a provoking factor for venous thromboembolism due to activation of coagulation pathways and endothelial injury [4]. However, the occurrence of PE following minor blunt trauma, in the absence of overt deep vein thrombosis (DVT) or conventional risk factors, is extremely rare and underreported in the literature. The clinical presentation of PE can also be nonspecific, often mimicking other cardiopulmonary or gastrointestinal conditions, which may contribute to delays in diagnosis and management [5]. Through this case, we aim to raise awareness of PE as a possible delayed complication of minor trauma and emphasize the importance of reassessment when symptoms persist or worsen.

## Case presentation

This case report describes a 55-year-old woman with a known history of cardiomegaly who developed a significant PE one week after sustaining seemingly minor blunt chest trauma. She had accidentally fallen onto the handlebars of a push-back bicycle, and her symptoms of sudden-onset epigastric pain and breathlessness began shortly afterward. Despite an initially normal D-dimer and CT scan, her condition progressed over time, ultimately requiring therapeutic anticoagulation.

On initial assessment, she was hemodynamically stable with a Glasgow Coma Scale (GCS) score of 15. Her vital signs included a respiratory rate of 19 breaths per minute, a heart rate of 76 beats per minute, and an oxygen saturation of 94% on room air. Physical examination revealed no signs of lower limb injury or clinical evidence of DVT. Systemic examination revealed tenderness in the epigastric region and lower ribs.

**Figure 1:**
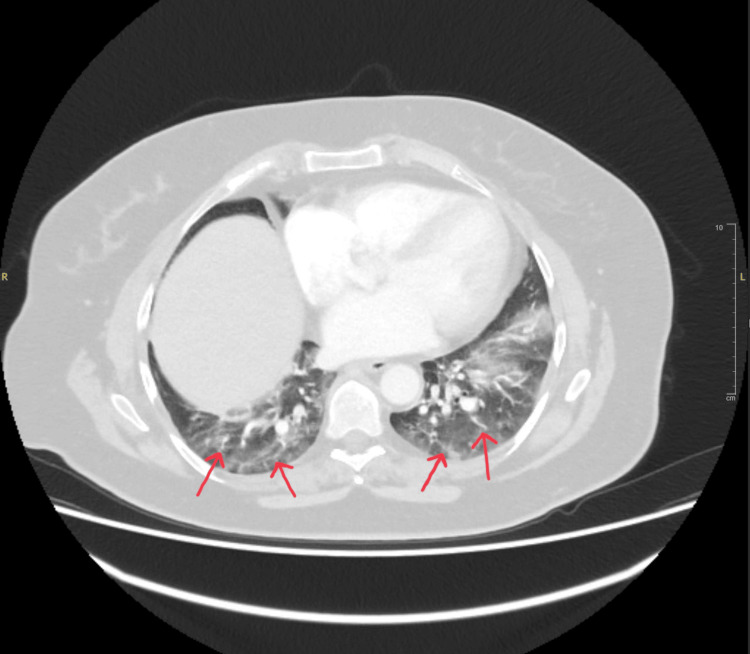
Cross-sectional CT of the chest and abdomen with contrast The red arrows indicate a significant dependent atelectatic change in both lungs with ground-glass attenuation in the lingula and both lower lobes.

The patient denied recent immobilization or personal or family history of thromboembolic disease and had no history of COVID-19 infection. Chest radiography demonstrated cardiomegaly without evidence of pneumothorax or rib fractures. The ECG showed a normal sinus rhythm with no repolarization changes. Initial laboratory investigations, including D-dimer, complete blood count, renal and liver function tests, and coagulation profile, were all within normal limits. The D-dimer was ordered because the patient presented with shortness of breath, in addition to epigastric pain and minor trauma. To exclude intra-abdominal injuries, a CT scan of the chest (Figure 1), abdomen, and pelvis was performed, revealing no acute abnormalities. She was discharged with advice for symptomatic management.

One week later, she re-presented to the emergency department with worsening dyspnea and pleuritic chest pain, accompanied by a productive cough with orange-tinged sputum. She remained normotensive with a GCS of 15. Her respiratory rate increased from 16-18 to 20 breaths per minute, and oxygen saturation fell to 88% on room air; supplemental oxygen at 2 L/min was initiated. Inflammatory markers, including WBCs, neutrophils, and CRP, remained normal.

**Figure 2:**
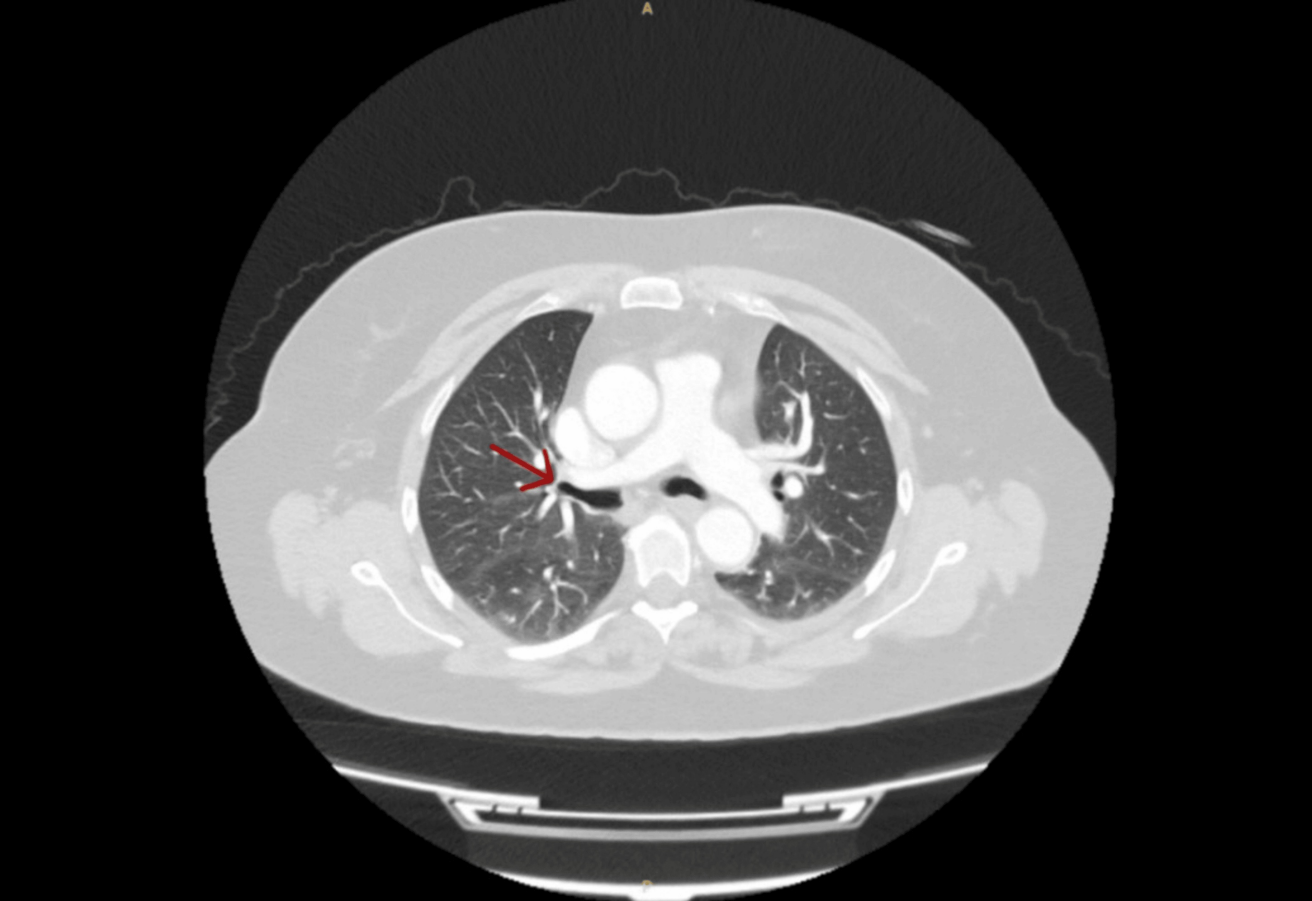
Cross-sectional CT of the chest and abdomen with contrast The red arrow indicates filling defects in the right inferior lobar and several right lower lobe segmental pulmonary artery branches, consistent with PE. Loss of lung volume with multifocal segmental atelectasis is more prominent compared to the previous examination. PE, pulmonary embolism

**Figure 3:**
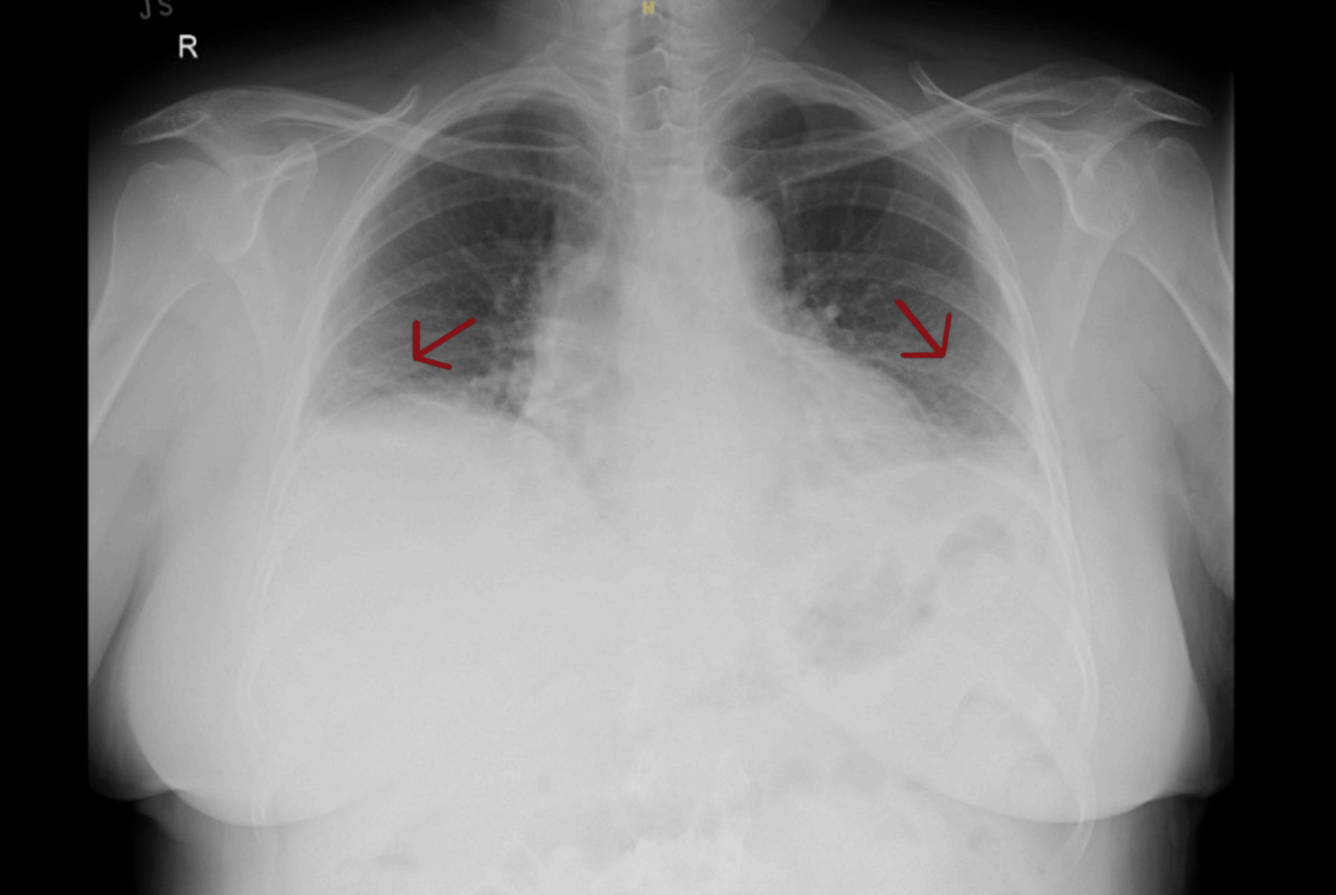
Chest X-ray showing bibasal hazy airspace opacification (red arrows), possibly representing infection An AP rather than a PA view was obtained due to the patient’s severe epigastric pain and shortness of breath, necessitating immediate imaging.

A repeat ECG showed a normal sinus rhythm. D-dimer levels were significantly elevated at 749 ng/mL, while other blood tests remained within normal limits. A CT pulmonary angiogram performed on her second visit (Figure 2) revealed filling defects in the right inferior lobar artery and several segmental branches, confirming PE. The scan also showed reduced lung volume and multifocal segmental atelectasis, which appeared more extensive than on her initial imaging. A follow-up chest X-ray (Figure 3) demonstrated bibasal hazy airspace opacification, possibly representing infection.

Given the absence of contraindications, therapeutic anticoagulation was promptly initiated with low molecular weight heparin (LMWH). The patient was admitted for monitoring and demonstrated clinical improvement over the next 24 hours. She was subsequently discharged on a direct oral anticoagulant (DOAC) with a planned treatment duration of three months and scheduled outpatient follow-up.

## Discussion

PE remains a critical concern, typically associated with venous thromboembolism, but it can also occur after minor trauma in the absence of traditional risk factors such as immobility, surgery, or thrombophilia [6]. Our case exemplifies this rare but important scenario: PE developing one week after minor chest trauma in a patient without typical predisposing factors and no laboratory evidence of infection, as inflammatory markers were within normal limits.

Trauma induces a hypercoagulable state through endothelial injury, systemic inflammation, and activation of coagulation pathways, consistent with Virchow’s triad, with an added inflammatory component [7]. Notably, PE can appear early after injury. Comprehensive reviews report incidences as high as 10-42% shortly after trauma in intensive care settings [8], and recent trauma cohort data confirm that PE can occur within the initial 72 hours [6].

In this case, the patient initially presented with epigastric pain and dyspnea but had normal D-dimer and CT scans, leading to conservative management. Symptoms worsened over a week, and re-evaluation revealed elevated D-dimer levels and lobar/segmental emboli on CT pulmonary angiography. This highlights the evolving nature of post-traumatic thrombus formation; initial evaluation may be misleading, and PE should be suspected when acute respiratory discomfort and significant upper abdominal pain develop [8].

This case underscores an important clinical lesson: initial negative investigations, even after trauma, do not rule out subsequent PE. When symptoms persist or worsen, repeating diagnostic tests, including D-dimer and imaging, can be lifesaving. Delayed presentations are well documented in trauma literature [8]. Regarding management, once PE was confirmed, anticoagulation with LMWH was promptly initiated, followed by transition to a DOAC. Guidelines for provoked venous thromboembolism, including trauma-associated events, recommend a treatment duration of at least three months [9,10], with recent American Society of Hematology guidance suggesting three to six months for most provoked events [11].

## Conclusions

PE following trauma can be challenging to diagnose, particularly in patients without traditional risk factors or clear evidence of DVT. This case demonstrates that even seemingly minor chest trauma can trigger a thromboembolic event. Importantly, a normal initial assessment should not rule out PE, especially if the patient’s condition deteriorates over time. Reassessment, including repeat imaging, may be necessary when symptoms progress. Prompt diagnosis and early initiation of anticoagulation remain critical for achieving favorable patient outcomes.
